# Supplementary material for: Chr15q25 Genetic Variant rs16969968 Alters Cell Differentiation in Respiratory Epithelia
Source: Int J Mol Sci. 2021 Jun 22;22(13):6657. doi: 10.3390/ijms22136657 (PMC8268843; doi:10.3390/ijms22136657)
Supplement: Supplementary file 1 [file ijms-22-06657-s001.zip › ijms-1261883-supplementary.pdf]

Supporting information

Chr15q25 genetic variant rs16969968 alters cell differentiation in respiratory epithelia

Zania Diabasana, Jeanne-Marie Perotin, Randa Belgacemi, Julien Ancel, Pauline Mulette, Claire Launois, Gonzague Delepine, Xavier Dubernard, Jean-Claude Mérol, Christophe Ruaux, Philippe Gosset, Uwe Maskos, Myriam Polette, Gaëtan Deslée, Valérian Dormoy

Table of content

1. Supporting information Tables 2

Table S1. List of primers. 2

Table S2. List of antibodies. 2

2. Supporting information Figures 3

Figure S1. CHRNA5 gene is expressed in the human airways 3

Figure S2. Cytokine expression at an early stage of differentiation 4

1. Supporting information Tables

Table S1. List of primers.

GENES	Forward sequence	Reverse sequence
**FOXJ1**	5’- CAGATCCCACCTGGCAGA-3’	5’- CGTACTGGGGGTCAATGC-3’
**CK5**	5’-TTCATGAAGATGTTCTTTGATGC-3’	5’-AGGTTGCGGTTGTTGTCC-3’
**MUC5AC**	5’- CACGTCCCCTTCAATATCCA-3’	5’- GGCCCAGGTCTCACCTTT-3’
**MUC5B**	5’- GTACAATGGCACCTTCTACGG-3’	5’- CTGACATTGCACCGTTGG-3’

Table S2. List of antibodies.

Antibodies	Species	Reference	Companies	Concentrations
**Foxj1**	Mouse	14-9965-82	Fisher Scientific	IF/IHC – 1:100
**Ki67**	Mouse	M7240	Agilent Dako	IF/IHC – 1:50
**Arl13b**	Rabbit	17711-1-ap	ProteinTech	IF/IHC – 1:200<br>WMIF – 1:200
**Uteroglobin**	Rabbit	10490-1-ap	ProteinTech	IF/IHC – 1:50
**P63**	Goat	AF1916	R&D systems	IF/IHC – 1:100
**Muc5b**	Rabbit	E-AB-15988	Elabscience	IF/IHC – 1:100<br>WMIF – 1:100
**Muc5ac**	Mouse	NBP2-15196	Novus Biological	IF/IHC – 1:100<br>WMIF – 1:100
**Vimentin**	Mouse	M0725	Agilent Dako	IF/IHC – 1:100
**CK13**	Goat	NBP-1 06047	Novus Biological	WMIF – 1:50

2. Supporting information Figures

Figure S1. CHRNA5 gene is expressed in the human airways

Representative micrograph showing α5SNP-coding mRNA expression (red dots) in bronchial epithelium. The slides were counterstained with hematoxylin (purple).

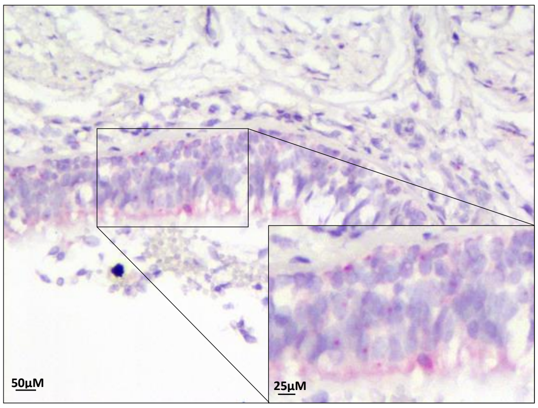

Figure S2. Cytokine expression at an early stage of differentiation

A. Table listing the position of control and cytokine capture antibodies on the membrane-based sandwich immunoassay. **B.** Examples of nitrocellulose membrane acquisitions displaying cytokine-associated spots. The chemoluminescence produced at each spot is proportional to the amount of cytokine bound. **C.** Table summarizing the normalized mean grey pixel values of cytokines and chemokines expression in α5WT- and α5SNP-expressing cells at ALI-7. The ratios α5SNP/α5WT are also presented.

A.

Position	Cytokine	Position	Cytokine	Position	Cytokine	Position	Cytokine
A1	Reference spot	B2	IL-1α	C4	IL-13	D6	MIF
A2	C5/C5a	B3	IL-1β	C5	IL-16	D7	MIP-1α
A3	CD40 Ligand	B4	IL-1ra	C6	IL-17	D8	MIP-1β
A4	G-CSF	B5	IL-2	C7	IL-17E	D9	Serpin E1
A5	GM-CSF	B6	IL-4	C8	IL-23	E1	Reference spot
A6	GR0α	B7	IL-5	C9	IL-27	E2	RANTES
A7	I-309	B8	IL-6	D2	IL-32α	E3	SDF-1
A8	SICAM-1	B9	IL-8	D3	IP-10	E4	TNF-α
A9	IFN-γ	C2	IL-10	D4	I-TAC	E5	sTREM-1
A10	Reference spot	C3	IL-12p70	D5	MCP-1	E10	Negative control

B.

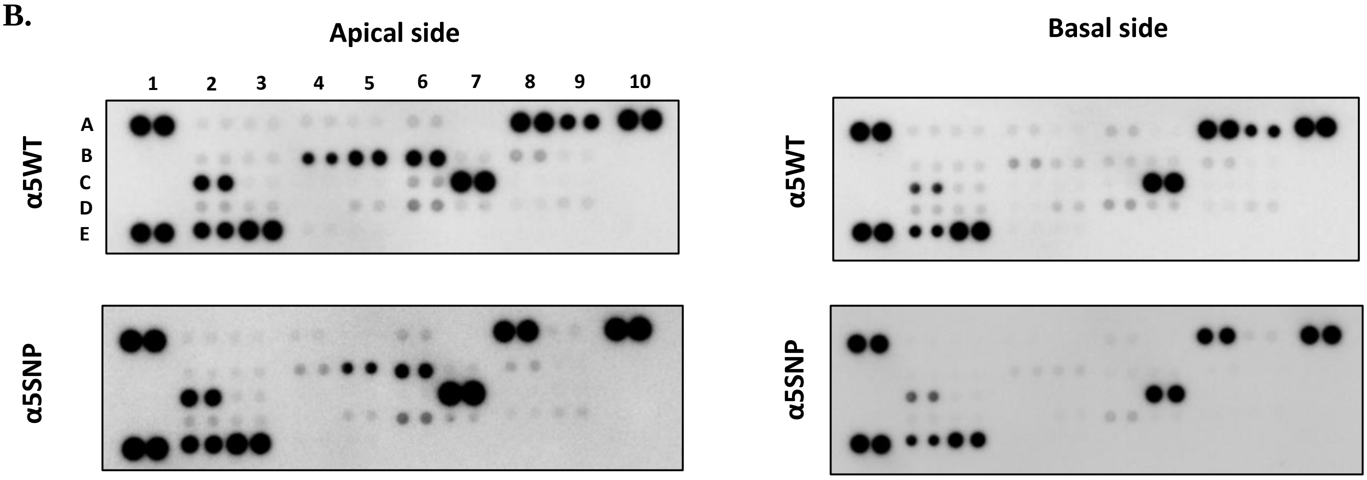

C.

	Apical side				Basal side			
**Cytokine**	**α5WT**	**α5SNP**	**Ratio**	**P-value**	**α5WT**	**α5SNP**	**Ratio**	**P-value**
C5/C5a	0,005	0,003	0,726	NA	0,005	0,003	0,657	NA
CD40 ligand	0,004	0,002	0,573	NA	0,004	0,002	0,471	NA
G-CSF	0,006	0,004	0,604	NA	0,007	0,003	0,402	NA
GM-CSF	0,007	0,004	0,567	NA	0,007	0,004	0,537	NA
GR0α	0,012	0,008	0,672	**0.013**	0,012	0,007	0,634	**<0.001**
I-309	0,003	0,002	0,886	NA	0,003	0,002	0,605	NA
IFN-γ	0,159	0,026	0,163	**<0.001**	0,046	0,019	0,417	**0.011**
IL-10	0,260	0,218	0,839	**0.001**	0,075	0,064	0,842	NS
IL-12 p70	0,007	0,004	0,577	**0.002**	0,006	0,004	0,688	**0.026**
IL-13	0,007	0,002	0,332	NA	0,005	0,003	0,655	NA
IL-16	0,007	0,004	0,595	NA	0,006	0,004	0,659	NA
IL-17	0,024	0,020	0,823	NS	0,016	0,013	0,815	NS
IL-17E	0,952	0,888	0,933	NS	0,802	0,646	0,806	NS
IL-1ra	0,103	0,063	0,604	NS	0,061	0,030	0,489	NS
IL-1α	0,011	0,008	0,733	NS	0,008	0,007	0,887	NS
IL-1β	0,014	0,011	0,765	NS	0,029	0,021	0,732	NS
IL-2	0,148	0,051	0,346	**<0.001**	0,043	0,011	0,260	**<0.001**
IL-23	0,001	0,002	1,149	NA	0,004	0,002	0,391	NA
IL-27	0,001	0,002	1,747	NA	0,004	0,001	0,228	NA
IL-32α	0,016	0,009	0,556	**<0.001**	0,012	0,008	0,658	**0.001**
IL-4	0,284	0,239	0,839	NS	0,035	0,021	0,592	NS
IL-5	0,014	0,009	0,667	**0.001**	0,012	0,009	0,756	**0.023**
IL-6	0,022	0,011	0,523	**<0.001**	0,011	0,005	0,464	**<0.001**
IL-8	0,006	0,003	0,504	NA	0,003	0,001	0,499	NA
IP-10	0,014	0,008	0,583	**<0.001**	0,011	0,008	0,706	**0.002**
I-TAC	0,006	0,002	0,323	NA	0,006	0,003	0,524	NA
MCP-1	0,011	0,005	0,469	**<0.001**	0,013	0,006	0,478	**<0.001**
MIF	0,021	0,012	0,565	**0.003**	0,019	0,011	0,593	**<0.001**
MIP-1α	0,011	0,014	1,330	NS	0,013	0,014	1,017	NS
MIP-1β	0,004	0,002	0,517	NA	0,004	0,002	0,531	NA
RANTES	0,303	0,241	0,796	**<0.001**	0,107	0,090	0,840	NS
SDF-1	0,692	0,552	0,797	**<0.001**	0,612	0,422	0,690	**<0.001**
Serpin E1	0,004	0,003	0,588	NA	0,004	0,002	0,452	NA
sICAM-1	0,569	0,518	0,910	NS	0,601	0,524	0,872	**0.009**
sTREM-1	0,007	0,003	0,383	NA	0,008	0,004	0,467	NA
TNF-α	0,010	0,003	0,292	NA	0,007	0,004	0,545	NA

NA: Not Available (protein detection below the threshold); NS: Non Significant
